# Strategic resilience in human performance in the context of science and education - perspective

**DOI:** 10.3389/fpsyt.2024.1410296

**Published:** 2024-04-24

**Authors:** Sabrina Ziehr, Philipp Hans Merkt

**Affiliations:** Unit of Applied Research 18_RECESS, Department of Disaster Prevention and Crisis Management Fresenius University of Applied Sciences, Idstein, Germany

**Keywords:** human performance, strategic resilience, special operating situations, problem-oriented intervention, mission success accomplished, 18_RECESS

## Abstract

**Introduction:**

Special situations that jeopardize the internal and external security for communities are increasing in their frequency and complexity. This creates complicated challenges for individuals, governments and humanity. National and international strategies are being developed that focus on the resilience and coping of all first responders during these extreme stress states.

**Aim:**

The aim of the article is to present the framework of strategic resilience, its multidimensional measurement and the possibilities for training robust resilience to increase operational effectiveness in special operational situations.

**Methodological approach:**

Research in the area of special operation situations often focuses on the human performance cluster. The Unit of Applied Research 18_RECESS (18_ Research and Education Center for Extraordinary Tactical Situations and Strategically Resilience) at the Department of Disaster Prevention and Crisis Management at Fresenius University of Applied Sciences in Idstein fits into this field. It pursues the adaptation of existing resilience models to special operation situations in line with the definition of strategic resilience. In addition, the focus is on the possibility of training strategic resilience to increase operational effectiveness. There are currently several research projects being conducted in the civilian and civilian-military sectors, as well as with ground and special operations forces.

**Major findings:**

Strategic Resilience with its 3 axes Psychological, Physiological and Cognitive Resilience covers the core domains of various existing models of resilience. This provides an adequate concept to describe different factors leading to personnel perseverance in special operation situations. There exists the possibility to train several domains of strategic resilience via problem-oriented intervention (POH).

## 1 Introduction

The demands on human performance in an emergency, generates high stress peaks for the professional groups of first responders. The human reaction to these stress peaks creates even more complexity within the coordinated response efforts between all official and non-official groups working together.

The interims between special situations that cause a “break in the usual routine at both an individual and systemic level” are becoming shorter (1). The intensity of crises and disasters appears to be increasing, as they are making themselves felt in ever larger sections of civilization. This creates the need for more diverse human performance and responses. In order to fulfil the state’s duty and ability to protect their citizens, the interaction of many first responders is required in times of peace and emergencies, crises and a state of national defense. The construct of Germany’s civil security and that of neighboring EU countries is increasingly being put to the test. Climate-related natural disasters (e.g. floods in the Ahr valley) are becoming more frequent, as are anthropologically induced emergencies (see, for example, the 11.5% increase in criminal offences in Germany from 2021 to 2022) or crises such as the Covid-19 pandemic, which have put civil security to the test (2). Furthermore, the Russian war of aggression against Ukraine has brought the issue of civil defense and civil protection back to the public’s attention. The resulting strain on the German security construct, i.e. internal and external disaster prevention, has an influence on the actors involved in disaster prevention. These actors include the Fire Service and the Federal Agency for Technical Relief (THW), which form an important part of Germany’s internal disaster prevention. The Fire Service is responsible for a wide range of disaster prevention tasks. They are responsible for firefighting, analyzing and averting CBRN dangers, providing first aid, rescuing people, and aiding in the event of major damage/natural disasters (3). In this way, they have an impact on civilian internal security, e.g. by maintaining critical infrastructures (3). The military in particular is frequently confronted with a wide variety of intense special operation situations that cause soldiers to be at a high risk for mental health disorders (4). However, special forces have a lower risk for mental health disorders compared to others of the military service (5). Killing someone leads to a higher risk for mental disorders, probably because of the divergence with the soldier’s moral code (4). Even the training of special deployment situations with the target group of humanitarian aid workers leads to a high level of stress among the participants, who are subsequently at an increased risk of PTSD (6). All named actors are confronted with a variety of security problems and stressors in the course of internal and external disaster prevention. In that context the development of (strategic) resilience to the described stressors is of great importance. The World Health Organization’s framework “Health Emergencies and Disaster Risk Management” (7), for example, which deals with research in the health sector, shows that resilience is a generally valid task for superordinate structures. However, the concept does not focus exclusively on the health sector but brings together different institutions such as public authorities and the business sector. At the national level in Germany, the importance of a cross-institutional view of resilience is emphasized by the federal government’s resilience strategy. This strategy considers all phases of disaster management at both an individual and societal level. This includes prevention, preparedness, coping and follow-up (8). Understood as a society-wide approach that requires knowledge from a wide range of different actors in order to strengthen resilience, this raises the question of how resilience can be measured and strengthened. Although a large number of different resilience models exist, such as Brand’s “Seven Pillars of Resilience” (9) or the “Core Life Skills” (10) or the health-related “Protective Factors of Resilience” (11) or Dinter’s “Star of Fortitude” (12) in the military field, the construct of resilience and its effects on robustness and performance have not yet been fully explored.

One field of research that has emerged in recent years, which examines robustness or performance in more detail (13), is the human performance cluster with its various sub-clusters (14):

Human Performance Modification - HPM: Modification of human performance to actively and passively change the performance level of a person. While Human Performance Modification usually focuses on optimization and improvement, HPM includes both degradation of human performance and recovery.
Human Performance Augmentation - HPA: Enhancement of human performance in science and technologies to temporarily or permanently improve conditioned human performance.
Human Performance Enhancement - HPE: Integration of existing and new methods in the context of pharmacological and neurochemical technology to the stimulation of human physiology and the biophysical capabilities of the individual.
Human Performance Degradation - HPD: Deterioration of human performance. Reduction of performance below previous levels resulting from biophysical capabilities: (I.) exhaustion, (II.) injury and illness, (III.) deterioration of psychological adaptive compliance.
Human Performance Restoration - HPR: Restoration of human performance. Return to baseline when performance has fallen below baseline. The focus is on restoring the reduction in the system triggered by (I.) exhaustion, (II.) injury and illness, (III.) deterioration in psychological adaptive compliance (15).
Human Performance Optimization - HPO: The process of applying existing and new science and technology to training in order to exceed a person’s biological outcome.

This is the field of research in which the Unit of Applied Research 18_RECESS at the Department for Disaster Prevention and Crisis Management at Fresenius University of Applied Sciences in Idstein works in. The different dimensions of resilience are at the center of this research: combining a wide range of disciplines including natural sciences, humanities and social sciences with medicine, psychology and spirituality. The aim of this research is to capture resilience multidimensionally and derive training options to increase the robustness of individuals in the context of human performance optimization in crises and disasters. Particular attention is paid to (special) forces, which play a key role in internal and external disaster prevention. To implement this research approach, 18_RECESS is working on internal projects and externally funded research projects. The internal research is concerned with the practical training courses offered within the Department of Disaster Prevention and Crisis. Participants in the “Tactical Operation Medicine - 18F” or “ Psychological Resilience - Land Survival - HEAT (18E)” (16) training courses undergo a series of tests before, during and after training programs to record individual indicators relating to resilience, e.g. biological parameters, physiological clinical parameters and cognitive tests (16) using sensory organs and psychosocial questionnaires or interviews. These measurements are also used in externally funded projects such as the research project: Psychological resilience research of air and ground-based emergency personnel to improve patient safety, currently ongoing with the DRF Stiftung Luftrettung gem. AG until 2026. The goal of this cooperation is the adaptation and integration of generally recognized resilience models in the context of human performance optimization in special operational situations. The first step is to develop and define a model that meets the requirements of special operational situations. This requires a precise representation of the individual axes and interfaces that influence robustness and performance in the context of human performance optimization. Operational effectiveness within a special operational situation depends on different factors. It can be assumed that the interplay of physical, cognitive and psychological factors play a particularly important role in the implementation of tactics and strategy (14). It can therefore be stated that a specific form of resilience is required of special operations forces who are confronted with special operational situations in their everyday duties: strategic resilience.

## 2 Strategic resilience

The term Strategic Resilience is primarily used in the military (17) and in business, especially since the coronavirus pandemic (18). In the business sector, Strategic Resilience tries to identify weak points, development potential and the conscious acceptance of risks. It represents a part of the St. Gallen management process and wants to implement it in the corporate strategy. For example, the Fraunhofer resilience cycle (Prepare-Prevent-Protect-Respond-Recover) finds use (18). Strengthening strategic resilience aims at better facing crises and emergencies in all management levels of the company. In the military, strategic resilience describes the approach of replacing the reactive nature of coping with major emergencies by dynamic situation assessments (17). First it aims to organize military operations proactively and thus avoid or reduce damage before happening by using dynamic leadership decisions (17).

Because of the increasing number of crises strategic resilience becomes more important. That fact has an impact on very different stages of life, concerning society, business and actors of internal or external disaster prevention. Resilience has to be improved for that all actors get more confident in facing prevention, management and follow up (7, 8). This requires a detailed picture of the situation and targeted decision-making embedded in a comprehensive strategy. Therefore it is crucial to examine strategic resilience independently of individual sectors. General models of resilience cannot capture that big picture, but give important directions. Based on these the authors deduce 3 subareas of strategic resilience in specific operational situations: Psychological - Physiological - Cognitive.

### 2.1 Psychological resilience

Psychological resilience deals with the emotional component of coping with a situation and describes a person’s personal mindset. Emotions can range from overwhelming to the inability of feeling emotions. The following aspects can be derived from the literature as sub-areas of psychological resilience:

- Optimism and hope as the ability to look positively into the future (19).
- Personal morality as character traits, attitude to life, positive emotions and drawing strength from religion and spirituality (12).
- Feeling involved, not feeling alone through a personal support network (11, 19).
- Self-efficacy and self-esteem as a source of strength in difficult situations (11, 12, 19).

In sum all sub-areas describe the personal mindset, depending on subjective feelings and attitudes. This restricts measurability because objective and standardized measurement methods are difficult to use. For this reason the investigation of psychosocial resilience depends on personal interviews completed by closed questionnaires.

### 2.2 Physical resilience

Physical Resilience describes the body functions that interact with stress in special situations. An important marker of physical resilience is the metabolic system. The recovery of the metabolic system is linked to the food intake, the existing stress level as well as to the circadian day-night rhythm. Results of research in nutrition seem to be promising despite the current lack of evidence (20). Furthermore it is discussed, that interruptions in sleep hygiene can have an impact on cognitive performance related to team leadership (21). The research team of 18_RECESS is investigating physical parameters during operational scenarios (22–24), the recruitment process for remote reconnaissance troops and the 24h operational flight of the DRF. Following parameters are examined:

Heart rate (continuous), blood pressure, body weight, body temperature, venous blood gases are obtained from the i-STAT ALINITY. Cg4: pH (pH units)+ PCO_2_ (mmHg) + PO_2_ (mmHg) + LACTATE (mmol/L). Chem8: Sodium (Na)+ Potassium (K)+ Chloride (Cl) + TCO2 + Anion Gap* + Ionised Calcium (iCa) + Glucose (Glu)+ Urea Nitrogen (BUN)/Urea Creatinine + (Crea)Hematocrit (Hct) + Hemoglobin* (Hgb).

Furthermore, cortisol and α-amylase are obtained using saliva samples and then analyzed using ELISA. Both cortisol, α-amylase and blood pressure are well suited to visibly describe the stress level and robustness. In addition to the laboratory parameters during the scenarios, the subjective assessment of the stress level is collected afterwards using a questionnaire (16, 24).

### 2.3 Cognitive resilience

Cognitive Resilience mostly depends on the performance in diverse physical and mental tasks under stress (25). Being cognitively alert in special operational situations is a mean factor of acting successful. Using the sensory organs defines the bridge between personal evaluation of the situation and the handling of incoming impressions. Misconnection between environment and personal estimation can cause a higher risk of failure. An explorative series of tests is used to measure the sensory organs: smell and taste, vision, hearing, touch and balance during stress-induced procedures up to 72 hours. The MARC test (24) is used for this purpose. Recognition (thresholds, sensitivity) and memory capacity are recorded. Cognitive sensory perception gains high consideration. Measuring follows a standardized protocol, starting with baseline to interpret followed by several measurements during stress.

### 2.4 The model of strategic resilience by Merkt & Ziehr

**Figure 1:**
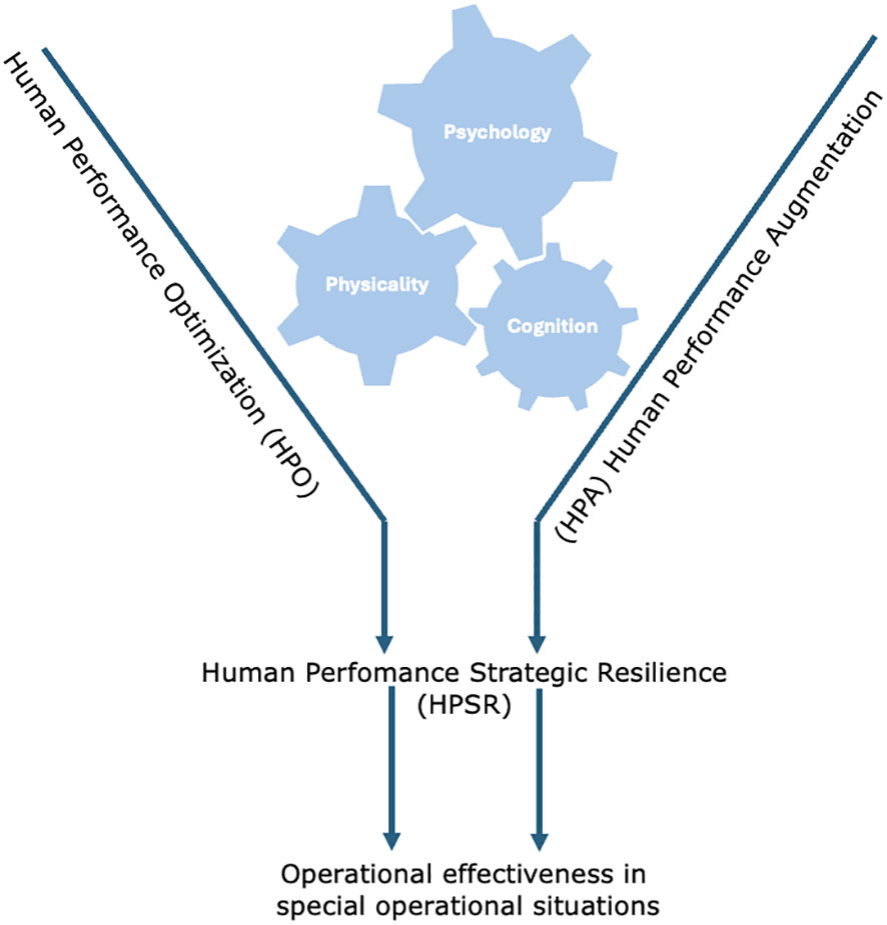
Model of strategic resilience in human performance by Merkt and Ziehr.

Merkt and Ziehr’s model of strategic resilience in human performance, as shown in **Figure 1**, centralizes the essential sub-areas of psychological, physical and cognitive aspects. It is eligible for specifically identifying relevant markers for personal capability to act and subsequent the operational effectiveness.

The capability to (re)act and the targeted decision making depends on the interaction between the 3 axes, that consolidate physical parameters, cognitive skills and the individual psychosocial factors.

## 3 Summary and discussion

In a world rife with crises that result in special operational situations (1), it has been recognized that resilience must be strengthened (7, 8). In special operational situations, however, common resilience models reach their limits. Examples of adapting resilience models to individual sectors such as the military or the economy exist. These include the strategic resilience approach that focus on overcoming crisis situations, preventing them, and making dynamic decisions (17, 18). The presented model of strategic resilience (Human Performance Strategic Resilience - HPSR) according to Merkt and Ziehr consolidates a multidimensional construct of resilience from different perspectives, such as the military, business and stress research. This allows a diverse group of first responders confronted with special operational situations to be considered. It is essential that the 3 axes of psychology, physicality and cognitive aspects are interlocked in a targeted manner to build the basis for developing a strategy that can be designed and pursued to improve operational effectiveness in special operational situations. The focused multidimensional consideration and strengthening of strategic resilience can help actors in basic and advanced training in official and non-official disaster prevention to improve their skills in terms of human performance optimization and human performance strategic resilience. That leads to a better outcome in special operational situations (life-threatening).

Exactly that is investigated by the authors’ research during the courses at the Department for Disaster Prevention and Crisis Management. All courses address people who are confronted with (life-threatening) special operational situations. Because of the various experts in theory, practice and science a high level in education is guaranteed so that targeted know-how can be provided. In order to ensure the transfer of theory to practice, the entire training program is scenario-based and a continuation of the already established method of problem-oriented learning (26). The authors name the method “Problem-oriented intervention (POH)” (24), similar to the problem-driven Research/Inquiry or Problem Based Synthesis or Project Based Learning (27). Participants are confronted with different situations they have to handle independently by situation assessment, exploration and evaluation. It takes place in a multi-professional team under the selective guidance and supervision of the teachers on site. First published research results show that the ability to intervene in special or complex operational situations can be increased through scenario-based education as part of the POH (23). It is shown, that all dimensions of individual strategic resilience are addressed:

- Physical Resilience: performance despite sleep deprivation and working under adverse conditions.
- Cognitive Resilience: gaining specialized knowledge and its adaption to the situation.
- Psychosocial Resilience: self-efficacy, e.g. by finding one’s own place in the team and working together in the team.

All together the factors lead to an increased confidence to (re)act (23). The increased confidence has a positive effect on robustness and the performance. Subsequently operational effectiveness is affected. For that reason the authors state, that tailored training improves Strategic Resilience in the concept of human performance optimization. Although these findings need to be investigated in more detail, they present a promising approach for the development and implementation of training programs for special operation situations in the context of human performance optimization.

## Data availability statement

The raw data supporting the conclusions of this article will be made available by the authors, without undue reservation.

## Ethics statement

The studies involving humans were approved by Ethikkommission der Hochschule Fresenius Idstein. The studies were conducted in accordance with the local legislation and institutional requirements. The participants provided their written informed consent to participate in this study.

## Author contributions

SZ: Conceptualization, Data curation, Formal analysis, Funding acquisition, Investigation, Methodology, Project administration, Resources, Software, Supervision, Validation, Visualization, Writing – original draft, Writing – review & editing. PM: Conceptualization, Data curation, Formal analysis, Funding acquisition, Investigation, Methodology, Project administration, Resources, Software, Supervision, Validation, Visualization, Writing – original draft, Writing – review & editing.
